# Prenatal Exposures to Common Phthalates and Prevalent Phthalate Alternatives and Infant DNA Methylation at Birth

**DOI:** 10.3389/fgene.2022.793278

**Published:** 2022-03-31

**Authors:** Rebekah L. Petroff, Vasantha Padmanabhan, Dana C. Dolinoy, Deborah J. Watkins, Joseph Ciarelli, Diana Haggerty, Douglas M. Ruden, Jaclyn M. Goodrich

**Affiliations:** ^1^ Department of Environmental Health Sciences, University of Michigan, Ann Arbor, MI, United States; ^2^ Department of Pediatrics, University of Michigan, Ann Arbor, MI, United States; ^3^ Department of Nutritional Sciences, University of Michigan, Ann Arbor, MI, United States; ^4^ Scholarly Activities and Scientific Support, Spectrum Health West Michigan, Grand Rapids, MI, United States; ^5^ Department of Obstetrics and Gynecology, Wayne State University, Detroit, MI, United States

**Keywords:** phthalates, DNA methylation, epigenetics, development, DOHAD

## Abstract

Phthalates are a diverse group of chemicals used in consumer products. Because they are so widespread, exposure to these compounds is nearly unavoidable. Recently, growing scientific consensus has suggested that phthalates produce health effects in developing infants and children. These effects may be mediated through mechanisms related to the epigenome, the constellation of mitotically heritable chemical marks and small compounds that guide transcription and translation. The present study examined the relationship between prenatal, first-trimester exposure of seven phthalates and epigenetics in two pregnancy cohorts (*n* = 262) to investigate sex-specific alterations in infant blood DNA methylation at birth (cord blood or neonatal blood spots). Prenatal exposure to several phthalates was suggestive of association with altered DNA methylation at 4 loci in males (all related to ΣDEHP) and 4 loci in females (1 related to ΣDiNP; 2 related to BBzP; and 1 related to MCPP) at a cutoff of *q* < 0.2. Additionally, a subset of dyads (*n* = 79) was used to interrogate the relationships between two compounds increasingly used as substitutions for common phthalates (ΣDINCH and ΣDEHTP) and cord blood DNA methylation. ΣDINCH, but not ΣDEHTP, was suggestive of association with DNA methylation (*q* < 0.2). Together, these results demonstrate that prenatal exposure to both classically used phthalate metabolites and their newer alternatives is associated with sex-specific infant DNA methylation. Research and regulatory actions regarding this chemical class should consider the developmental health effects of these compounds and aim to avoid regrettable substitution scenarios in the present and future.

## Introduction

Phthalates are a commonplace class of chemicals that are found in a sundry of modern consumer, building, and medical products. This diverse group of chemicals are used in plastic products as additives to increase their durability and flexibility, as well as in soaps and other personal care products as solvents. In everyday life, people are exposed to phthalates through the inhalation of phthalate dust from products like vinyl flooring, through incidental dietary exposure from contaminated dairy goods or food-packaging materials, and through dermal absorption from the direct use of soaps, fragrances, and make-up (Graham, 1973; Braun et al., 2013). Importantly, these chemicals readily leach out of their products, and, because of their endemic nature, nearly every person in the United States has detectable levels of one or more phthalate metabolites in their urine (Woodruff et al., 2011; Zota et al., 2014).

While phthalates are widely known for their antiandrogenic effects (Qian et al., 2020), particularly after high-dose exposure in males (>0.09 mg/kg/day, see Taiwan poisoning) (Li and Ko, 2012; Yang et al., 2013), strong evidence from both human and animal models has increasingly demonstrated that lower levels of exposure are related to a number of endocrine, reproductive, metabolic, and neurological health outcomes (Benjamin et al., 2017; Braun, 2017; Radke et al., 2019; Radke et al., 2020; Engel et al., 2021), These effects are not evident until later in life, but have been linked to early-life exposure to phthalates, in accordance with the Developmental Origins of Health and disease (DOHaD) hypothesis (Barker, 2007).

One mechanism by which the DOHaD hypothesis has been posited to operate is via alterations to the epigenome, the mitotically heritable collection of chemical marks and other molecules that regulate gene transcription and translation (Barouki et al., 2018). A commonly studied aspect of the epigenome is the DNA methylome, which is comprised of small methyl groups that typically bind to cytosine residues upstream of guanine residues (CpG sites) in the DNA, potentially altering chromatin structure and DNA transcription (Jones, 2012). Importantly, in the first few weeks of development, the fetal methylome is erased and rewritten, so exposures during this critical window can impact the lifelong epigenome of an individual (Bacchelli and Bollati, 2009; Perera et al., 2020).

Some studies in humans have demonstrated that developmental phthalate exposure is connected with an altered epigenome in early life, and scientists have subsequently suggested that these chemicals can have a significant impact on the epigenome and associated long-term health (LaRocca et al., 2014; Wang I.-J. et al., 2015; Goodrich et al., 2016; Huen et al., 2016; Solomon et al., 2017; Chen et al., 2018; Huang et al., 2018; Montrose et al., 2018; Tindula et al., 2018; Miura et al., 2021). Most of these studies, however, utilized targeted techniques to examine how methylation in a small number of genes and genetic elements is associated with phthalate exposure. Three studies used a genome-wide approach (Illumina 450K array) to quantify DNA methylation in cord blood and reported different sets of enriched genes, which shared some common pathways (e.g., those in endocrine, metabolism) that were associated with phthalates (Solomon et al., 2017; Chen et al., 2018; Miura et al., 2021). These studies differed by the timing of exposure assessment during pregnancy (from early to late gestation) and the phthalates included; two studies only included metabolites of one particular phthalate (DEHP) (Chen et al., 2018; Miura et al., 2021). Further, phthalate use and exposures are rapidly changing (Zota et al., 2014). There is little research on how newer phthalates and phthalate alternatives may impact the epigenome of developing infants.

The current manuscript builds on the body of phthalate research by combining data from two pregnancy cohorts based in Michigan to assess associations between prenatal exposure to seven common phthalates (metabolites of DEHP, DEP, DiNP, DnBP, DiBP, BBzP, and the multi-parent compound metabolite MCPP) and sex-specific infant DNA methylation at birth (in cord blood or neonatal blood spots). Expanded analyses in a subset of these dyads also explore whether two compounds that are increasingly used as alternatives to phthalates, DINCH and DEHTP, are associated with infant DNA methylation (in cord blood).

## Materials and Methods

### Study Population

Data from this study were collected from two pre-birth cohorts based in the state of Michigan: the Archives for Research on Child Health (ARCH) cohort (Slawinski et al., 2018) and the Michigan Mother-Infant Pairs (MMIP) cohort (Goodrich et al., 2019). Pregnant people with singleton pregnancies and who were at least 18 years old were recruited for each cohort. All participants gave informed consent prior to participating in the study and procedures were approved through the Institutional Review Boards at Michigan State University and the Michigan Department of Health and Human Services (ARCH, Approval: LEGACY 16-1429M) and the University of Michigan Medical School (MMIP, Approval: HUM00017941). For the current study, parent-infant dyads with first-trimester, urinary phthalate metabolite exposure measurements and DNA methylation data from infant birth samples were included.

In the first trimester (10–14 weeks), those enrolled in ARCH were surveyed and provided a urine sample for phthalate metabolite analysis. At delivery, dried blood spots were collected for newborn screening and leftover blood spots were made available for this research through the Michigan BioTrust for Health program. Similarly, MMIP participants gave survey data and urine during their first-trimester visit (8–14 weeks). At delivery, umbilical cord blood samples were collected into PaxGene Blood DNA tubes (Preanalytix, Hombrechtikon, Switzerland). Samples were stored at −80°C until analysis.

### Demographic and Clinical Covariates

Variables ascertained from ARCH and surveyed from MMIP included ethnicity and race, marital status, smoking history, and household income. Clinical data collected included the route of delivery, infant’s gestational age, sex, and birthweight, as well as weight in early pregnancy and post-delivery, height, and parental age at pregnancy start. Body mass index (BMI) was calculated from these values.

### Phthalate Metabolite Measurements

The exposures of interest for the present study included the metabolites of six parent phthalates and one nonspecific metabolite, quantified in urine, that were shared between ARCH and MMIP (see Table 1 for details). Urine samples from ARCH were collected using the clean-catch method, stored for up to 24 h at 4°C before being aliquoted and then stored at −80°C until analysis. Urinary phthalate metabolites were analyzed using enzymatic deconjugation, solid-phase extraction, and high-performance liquid chromatography-electron spray ionization (HPLC-ESI) mass spectrometry, as previously described (Haggerty et al., 2021). MMIP urine was similarly collected and stored at −80°C until analysis. Based on Centers for disease Control and Prevention (CDC) laboratory methods, isotope dilution liquid chromatography was used to measure urinary phthalate metabolite concentrations in three separate batches, as previously described (Watkins et al., 2016; Rocha et al., 2017; Goodrich et al., 2019).

**TABLE 1:** Phthalate metabolite concentrations in first-trimester maternal urine samples.

Parent Exposure	Urinary Metabolite	% Below Limit of Detection			Dilution-Corrected Geometric Mean Concentration ± GSD (Range, Corrected-ng/ml)			
		ARCH (n = 128)	MMIP (n = 134)	All ARCH (n = 128)		All MMIP (n = 134)	Combined Females (n = 114)	Combined Males (n = 148)
ΣDEHP (di(2-ethylhexyl) phthalate)				40.18 ± 2.3 (6.5–871.3)		28.74 ± 2.7 (1.3–1614.9)	31.84 ± 3.2	28.22 ± 2.9
	MEHP (mono(2-ethylhexyl) phthalate)	43.8	15.5	1.19 ± 3.7 (0–113.1)		1.80 ± 2.2 (0–75.8)	1.93 ± 3.2	1.18 ± 3.0
	MEOHP (mono(2-ethyl-5-oxohexyl) phthalate)	0	0	3.66 ± 2.4 (0.5–64.4)		3.30 ± 2.6 (0.2–165.8)	3.32 ± 2.9	2.86 ± 2.8
	MEHHP (mono(2-ethyl-5-hydroxyhexyl) phthalate)	0	0	6.59 ± 2.5 (0.9–139.1)		10.00 ± 2.9 (0.4–716.7)	8.00 ± 3.0	6.61 ± 3.1
	MECPP (mono(2-ethyl-5-carboxypentyl) phthalate)	0	0	10.46 ± 2.3 (1.6–175.8)		6.69 ± 2.6 (0.3–265.0)	7.69 ± 3.3	7.04 ± 3.0
	MCMHP (mono(2-carboxymethylhexyl) phthalate)	0	NA	7.62 ± 2.5 (1.3–172.0)			9.18 ± 2.8^a^	5.66 ± 2.7^b^
DEP (di-ethyl phthalate)								
	MEP (mono-ethyl phthalate)	0	0	51.24 ± 4.0 (6.3–6773.2)		21.16 ± 3.7 (0.6–1042.2)	33.03 ± 4.8	25.67 ± 4.8
ΣDiNP (di-isononyl phthalate)				6.23 ± 3.1 (0.7–98.2)		4.04 ± 3.75 (0–237.7)	5.29 ± 4.5	4.17 ± 3.9
	MCiOP^c^ (mono(2,6-dimethyl-6-carboxyhexyl) phthalate)	1.6	9	4.42 ± 3.2 (0.4–70.4)		2.63 ± 3.6 (0–182.0)	3.69 ± 4.4	2.78 ± 3.9
	MCiNP^c^ (mono-carboxy-isononyl phthalate)	19.5	35.8	0.22 ± 3.4 (0–5.2)		0.85 ± 2.3 (0–21.8)	0.60 ± 3.3	0.34 ± 4.2
	MiNP (mono-isononyl phthalate)	NA	94.1			^d^	^d^	^d^
DiBP (di-isobutyl phthalate)								
	MiBP (mono-isobutyl phthalate)	0	0.7	5.15 ± 2.1 (1.1–67.1)		4.69 ± 2.8 (0–154.5)	4.83 ± 2.8	4.17 ± 2.9
DnBP (di-n-butyl phthalate)								
	MnBP (mono-n-butyl phthalate)	0	0	8.56 ± 1.9 (2.2–53.6)		7.55 ± 2.3 (0.6–132.2)	7.99 ± 2.8	6.41 ± 2.5
BBzP (butyl benzyl phthalate)								
	MBzP (mono-benzyl phthalate)	0.8	1.5	10.20 ± 2.5 (1.1–102.8)		3.54 ± 2.8 (0–182.0)	6.07 ± 3.6	4.89 ± 3.7
Nonspecific								
	MCPP (mono-(3-carboxypropyl) phthalate)	0.8	5.2	1.80 ± 2.5 (0.3–38.4)		1.65 ± 2.6 (0–30.74)	1.76 ± 3.2	1.47 ± 3.1
**MMIP-only Exposures**						**n = 79**	**n = 38**	**n = 41**
ΣDINCH (cyclohexane-1,2-dicarboxylic acid-diisononyl ester)						0.80 ± 1.7 (0–43.0)	0.85 ± 1.7	0.88 ± 1.4
	MCOCH (cyclohexane-1,2-dicarboxylic acid-monocarboxy isooctyl ester)	NA	6.3			0.92 ± 1.5 (0–7.2)	0.98 ± 1.1	0.92 ± 1.4
	MHNCH (cyclohexane-1,2-dicarboxylic acid-mono(hydroxy-isononyl) ester)	NA	6.2			0.70 ± 1.9 (0–25.0)	0.73 ± 1.7	0.79 ± 1.6
ΣDEHTP (di(2-ethylhexyl) terephthalate)						8.09 ± 5.6 (0–1019.1)	7.53 ± 5.2	6.14 ± 6.7
	MECPTP (mono-2-ethyl-5-carboxypentyl terephthalate)	NA	0			5.76 ± 5.5 (0–709.6)	5.31 ± 5.1	4.41 ± 6.5
	MEHHTP (mono-2-ethyl-5-hydroxyhexyl terephthalate)	NA	0			1.28 ± 3.5 (0–97.8)	1.10 ± 3.2	1.23 ± 4.3

NA, not measured in that cohort.

a n = 46.

b n = 82.

c Also a known metabolite of DDP (diisodecyl phthalate).

d Not reported as individual concentration due low number above the limit of detection.

For metabolites in ARCH, creatinine concentrations, measured with HPLC (Shimadzu LC-30 CE Series HPLC system, Shimadzu Corporation, Kyoto, Japan/Sciex 5500, ESI-MS/MS; Applied Biosystems, Foster City, CA) were used to adjust for urinary dilution, as described as Kuiper et al. (2021). For ARCH, raw concentrations were input into the following formula:

$$\mathrm{Raw}\;\mathrm{Concentration}*\frac{\mathrm{Median}\;\mathrm{Cohort}\;\mathrm{Creatinine}}{\mathrm{Individual}\;\mathrm{Creatinine}}$$

For MMIP, urinary specific gravity, measured using a handheld device (ATAGO Company, Ltd, Tokyo, Japan), was used to adjust concentrations for urinary dilution. MMIP dilution-corrected concentrations were calculated using the following formula:

$$\mathrm{Raw}\;\mathrm{Concentration}*\frac{\mathrm{Median}\;\mathrm{Cohort}\;\mathrm{Specific}\;\mathrm{Gravity}-1}{\mathrm{Individual}\;\mathrm{Specific}\;\mathrm{Gravity}-1}$$

In both cohorts, any concentration that was measured below the limit of detection (LOD) was imputed with LOD/ √2 . Non-detected observations were treated as 0. Phthalates measured from both cohorts include the parent compounds DEHP (secondary metabolites: MEHP, MEOHP, MEHHP, MECPP, and MCMHP), DEP (secondary metabolite: MEP), DiNP (secondary metabolites: MCiOP, MCiNP, and MiNP), DiBP (secondary metabolite: MiBP), DnBP (secondary metabolite: MnBP), and BBzP (secondary metabolite: MBzP), as well as the non-specific phthalate metabolite MCPP. For parent phthalates with more than one secondary metabolite (summed DEHP or ΣDEHP; summed DiNP or ΣDiNP), individual metabolites were summed by calculating the molar sums for each using the formula:

$$\mathrm{Parent}\;\mathrm{Molecular}\;\mathrm{Weight}\times \left(\frac{\mathrm{Concentratio}n_{\mathrm{Metabolite}\;A}}{\mathrm{Molecular}\;\mathrm{Weigh}t_{\mathrm{Metabolite}\;A}}+\frac{\mathrm{Concentratio}n_{\mathrm{Metabolite}\;B}}{\mathrm{Molecular}\;\mathrm{Weigh}t_{\mathrm{Metabolite}\;B}}\ldots +\frac{\mathrm{Concentratio}n_{\mathrm{Metabolite}\;n}}{\mathrm{Molecular}\;\mathrm{Weigh}t_{\mathrm{Metabolite}\;n}}\right)$$

In addition to the set of phthalates that were common across both cohorts, two parent phthalate alternatives of increasing scientific interest, DEHTP and DINCH, were also measured in one set of MMIP samples. Concentrations below the LOD were imputed with LOD/ √2 , and log-transformed, molar summed exposures for these two parent compounds were calculated from secondary metabolite concentrations, using the above formula (MCOCH and MHNCH for ΣDINCH; MECPTP and MEHHTP for ΣDEHTP). Any parent compound with less than 60% of individuals with all metabolite values below the LOD was removed from analysis.

### DNA Isolation and Methylation Conversion

From either blood spots (ARCH) or cord blood (MMIP) collected at birth, the EZ1 DNA Investigator Kit (Qiagen, Germantown, MD) and the PaxGene Blood DNA kit, respectively, were used to extract total genomic DNA. Extracted DNA was then bisulfite converted with a DNA methylation kit (Zymo Research, Irvine, CA). DNA methylation was then quantified at >850,000 CpG sites via the Infinium MethylationEPIC (Moran et al., 2016) (the “EPIC,” Illumina Inc., San Diego, CA), following the suggested protocols. The ARCH analysis was performed at Wayne State University, and the MMIP analysis was completed by the University of Michigan Advanced Genomics Core as previously described (LaBarre et al., 2020; McCabe et al., 2020).

### Epigenetic Data Quality Control

EPIC data quality control and preprocessing were conducted in R (version 4.02) (Team, 2020), with BioConductor packages Enmix (Xu et al., 2016) and minfi (Aryee et al., 2014; Fortin et al., 2016). Quality control procedures included sex prediction based on X and Y chromosome methylation, evaluation of bisulfite conversion efficiency, and number of failing probes in each sample. Any sample was removed in which predicted sex did not match recorded sex, conversion efficiency was poor, or >5% of probes failed detection. In total, 43 samples from ARCH and 27 samples from MMIP failed quality control and were excluded, giving final sample sizes of 128 and 134, respectively. In MMIP, a subset of samples were duplicated. For any duplicated samples, a sample that passed quality control was randomly selected for inclusion in the statistical analysis.

Probes that were in SNP regions, known to be cross-reactive (Pidsley et al., 2016), were not detected in >5% of samples (*p*-value > 1E-10 compared to background), were differentially measured in duplicated samples (>5% difference in methylation), or were in the X and Y chromosomes were excluded from the analysis in both cohorts. Approximately 82% (n = 708,641) of probes were included in ARCH and 88% (n = 766,302) of EPIC probes were included in MMIP, yielding a total of 706,492 shared probes across 262 dyads in both cohorts. For MMIP-only analyses of ΣDINCH and ΣDEHTP, 79 pairs were included in analyses, with 763,541 probes. In samples passing these criteria, noob background and dye bias corrections were applied using RELIC (Xu et al., 2017) in Enmix, and methylation was normalized within each cohort, using Enmix’s quantile normalization. Cell type proportions in each sample were estimated from an established algorithm based on reference data from sorted cord blood cells (Bakulski et al., 2016).

### Statistical Analysis

All statistical analyses were conducted in R. Geometric means and distributions of concentrations were calculated for each exposure. Log-transformed, dilution-corrected exposure concentrations were used in all statistical analyses. Categorical and continuous demographic variables shared between cohorts were assigned the appropriate category or converted to the same units to match scales between cohorts. Missing values were imputed with the median of both cohorts. Descriptive statistics for demographics and exposures were calculated and compared between cohorts using standard t-tests or chi-square tests as appropriate.

Singular Value Decomposition (SVD) analysis was performed with the R ChAMP package (Tian et al., 2017) to identify technical and biological covariates that correlate with variation in DNA methylation data. The correlation between methylation principal components and covariates was determined using linear regression for continuous variables or Kruskal–Wallis for categorical variables. Covariates considered included cohort (ARCH or MMIP), maternal variables (marriage status, income, age, BMI), infant variables (birth route, birthweight, sex, estimated cell type proportions), and technical variables. Surrogate variables, accounting for unmeasured or unknown sources of noise, were calculated from control probe data amongst all individuals in ARCH and MMIP to represent technical variation (Teschendorff et al., 2011).

Infant sex-stratified, multivariate linear regression was used to examine the relationship between first-trimester phthalate exposure and infant blood methylation at each individual CpG site (modeled as beta values representing the proportion methylated from 0 to 1), while controlling for common confounding variables. Confounding variables included in the models were cohort, maternal BMI in early pregnancy, maternal income (dichotomized at a cutoff of $50,000), infant blood cell type proportions (granulocytes, CD8^+^ T cells, B cells, and nucleated red blood cells), and the surrogate variables described above. An empirical Bayesian moderation method from the R package limma as used to shrink probe-wise variances towards a pooled estimate prior to significance calling (Smyth et al., 2005). A *q*-value of 0.2 was used to identify CpG sites that are suggestive of significance, and a stricter *p*-value cutoff of 9E-8 was used to call highly significant differentially methylated CpG sites (Mansell et al., 2019).

For MMIP-only analyses of ΣDINCH and ΣDEHTP, a similar linear model with a Bayesian moderation was used to regress the individual CpG site methylation on exposure, controlling blood cell type proportion (granulocytes, CD8^+^ T cells, B cells, and nucleated red blood cells), as well as surrogate variables accounting for unmeasured or unknown sources of noise on the MMIP cohort only. Initially, sex-interaction models were used to examine significant CpG loci and sex-stratified models were used to explore pathways, as the sample size in this smaller set of MMIP-only individuals did not allow for sex-stratification analyses (see next section for details). Lambda values (genomic inflation factor) were calculated for every model to estimate the level of *p*-value inflation or deflation. A *q*-value of 0.2 was used to identify initial CpG sites suggestive of significance, and *p*-value cutoff of 9E-8 was considered for highly significant sites (Mansell et al., 2019).

### Ingenuity Pathway Analysis

For each of these sex-stratified models of phthalate or phthalate alternative exposure, CpG sites with a *p*-value < 0.001 were annotated to a known gene and were uploaded to Qiagen’s Ingenuity Pathway Analysis (IPA) software (Qiagen). IPA uses known and predicted relationships between regulators and their target genes in the Ingenuity Knowledge base to test significant relationships in the dataset of interest. A given CpG site can be mapped to more than one gene, and more than one CpG site can be mapped to multiple genes. In IPA, the single most significant CpG for a given gene is considered; other CpG sites for the same gene are removed from analysis. The Tox Analysis function was used to assess the 1) canonical pathways, 2) Tox Lists, and 3) general molecular functions that were significantly enriched in this dataset and predict gene lists that were significantly enriched. These select functions from IPA have extensive databases that can give additional insight into potential relationships underlying the top genes that were differentially methylated. Importantly, the canonical pathways and molecular functions consider not only the genes in the set, but also the effect size (*β* values). Tox lists strictly consider the genes present, so are considered exploratory and hypothesis generating. To assess sources bias from multiple CpG sites mapped to a single gene, we also compared these results to those from a random set of genes representing the average number of genes across all models, using *β* values from a randomly selected model (in this case, Male-BBzP). Human pathways or gene lists enriched with a *p*-value < 0.05 and a *z*-score > 2 or < −2 were considered significant. Because this analysis was conducted with methylation data, which typically relates increases in methylation to decreasing gene expression, relationships designated as significantly inhibited (less methylated) in the software are considered to be activated in the present manuscript. Similarly, significantly activated (more methylated) relationships could be considered to be downregulated.

## Results

### Demographics and Average Exposures

**FIGURE 1:**
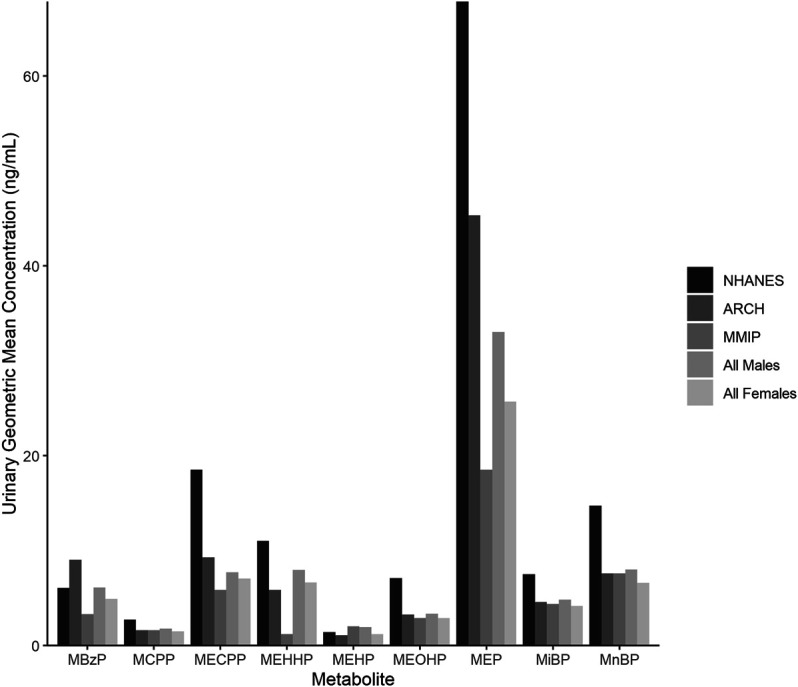
Phthalate metabolite concentrations in urine from the National Health and Nutrition Examination Survey (NHANES), ARCH, and MMIP. Each bar represents the geometric mean of metabolites measured in NHANES, ARCH, and MMIP, as well as the combined ARCH and MMIP cohorts by infant sex.

Maternal characteristics varied somewhat between cohorts (Table 2). In general, most mothers self-reported as white (ARCH: 78.1%; MMIP: 83.7%) and never-smokers (ARCH: 84.4%; MMIP 77.6%). Average BMI was higher in ARCH (28.92) compared to MMIP (25.45). Average marital status and income were also different between cohorts. In ARCH, 45.3% mothers reported being married, compared with over 80% of MMIP mothers. For income, 78.1% of ARCH mothers had reported income below $50,000, whereas 75.4% of MMIP mothers were reported to be above $50,000. Route of delivery was not statistically different between cohorts; over 70% of infants from both cohorts were born vaginally. Gestational age, however, was significantly different between cohorts (ARCH: 38.8 weeks, MMIP: 39.6 weeks), as was sex distribution. ARCH infants slightly skewed towards male (64.1%), whereas MMIP infants were evenly divided between males (49.3%) and females (50.1%). Birthweights were similar between cohorts (ARCH: 3.33 kg; MMIP: 3.45 kg). Phthalate metabolites were well detected in both cohorts, with most metabolites being measured in the majority of participants (Table 1). After correction for urinary dilution, ARCH and MMIP concentrations had similar means and ranges. ΣDEHP (specifically the MECPP metabolite) and DEP were found in some of the highest concentrations, but most other metabolite concentrations and ranges were comparable between cohort and between national measures of phthalate exposure (see Figure 1 for comparisons with adult female phthalate metabolites from the National Health and Nutrition Examination Survey (NHANES) [Center for Disease Control and Protection (CDC), 2021)]. Males, on average, had higher concentrations of most exposures than females, but most differences were minimal (Table 1).

**TABLE 2:** Summary of cohort demographics.

Reported as mean and SD or %			ARCH (n = 128)	MMIP (n = 134)	*p*-value
**Maternal Characteristics**					
Age (years)			26.79 (5.4)	32.02 (4.0)	<0.001
Race and Ethnicity					
		Asian	3.9%	3.0%	
		Black	15.6%	6.7%	
		White	78.1%	83.7%	
		Hispanic	12.5%	2.2%	
		Other	2.4%	6.0%	
Income					
		<$25,000	57.8%	14.2%	<0.001
		$25,000 to $49,000	20.3%	8.2%	
		$50,000 to $74,999	8.6%	19.4%	
		>$75,000	11.7%	56.0%	
Marital Status					
		Married	45.3%	82.1%	<0.001
		Single	53.9%	17.2%	
Any Smoking					
		No	84.4%	77.6%	0.97
		Yes	12.5%	16.3%	
		Early Pregnancy BMI (kg/m^2^)	28.92 (8.3)	25.45 (5.7)	<0.001
**Infant Birth Characteristics**					
Route of Delivery					
		Vaginal	71.9%	73.1%	0.93
		Cesarean	28.1%	26.9%	
Gestational Age (weeks)			38.81 (1.4)	39.59 (1.2)	<0.001
Sex					
		Male	64.1%	49.3%	0.02
		Female	35.9%	50.7%	
Birthweight (kg)			3.33 (0.5)	3.45 (0.5)	0.06

### DNA Methylation and Phthalates: Main Results

Using the 262 dyads (*n* = 148 male infants; *n* = 114 female infants) from two cohorts and methylation data from 706,492 EPIC probes, regression models were fit for six parent phthalates and one nonspecific metabolite. Across all exposures, regression model lambdas were sufficiently minimized, although several were slightly inflated in females (Supplementary Table S1). In males, DNA methylation at 4 CpG sites were associated with ΣDEHP exposure (Table 3). Methylation at one locus was increased with increasing exposure, a locus in the forkhead box protein 4 (*FOXP4*) gene (*β* = 0.002, *q* = 0.009), which also met the statistical threshold of *p* = 9E-8. Three loci were suggestive of having decreased methylation in relation to ΣDEHP exposure, including loci in the NOD-like receptor P3 (*NLRP3*) gene (*β* = −0.005, *q* = 0.080), the (*ZNF169*) gene (*β* = −0.013, *q* = 0.080), and an unidentified region on chromosome (*β* = −0.004, *q* = 0.080). In females, another 4 loci were suggestive of having decreased methylation in association several different exposures (Table 3). These included one locus associated with ΣDiNP in the dynamin 2 (*DNM2*) gene (*β* = −0.003, *q* = 0.076); two loci associated with BBzP in a zinc finger gene (*ZMIZ1*; *β* = -0.024, *q* = 0.14) and a zinc transporter gene (*SLC39A10*; *β* = -0.003, *q* = 0.076); and one locus associated with MCPP in the thiamine triphosphatase (*THTPA*) gene (*β* = −0.018, *q* = 0.061). None of these CpG sites in females were significant at a strict *p*-value cutoff of 9E-8. Notably, the cohort/sample type were well-distributed across these relationships and coefficients of CpG sites (*q <* 0.2) were not well correlated between male and female models, suggesting that the associations between phthalate metabolites and DNA methylation are dissimilar between sexes at many CpG sites.

**TABLE 3:** CpG loci suggestive of association (*q* < 0.2).

Exposure	CpG Illumina ID	Chromosomal Location	Gene	CpG Position Relative to	Coefficient	SE	*p*-value	*q*-value
Male Infants								
ΣDEHP	cg18497508	chr6:41528345	*FOXP4*	Island	0.002	0.000	1.26E-08	8.92E-03
ΣDEHP	cg10984680	chr4:118009982		S_Shelf	-0.004	0.001	3.30E-07	8.01E-02
ΣDEHP	cg16793775	chr19:56469964	*NLRP8*	OpenSea	-0.005	0.001	4.11E-07	8.01E-02
ΣDEHP	cg03808528	chr9:97060684	*ZNF169*	OpenSea	-0.013	0.003	4.53E-07	8.01E-02
Female Infants								
ΣDiNP	cg02688142	chr19:10893462	*DNM2*	OpenSea	-0.003	0.001	1.07E-07	7.57E-02
BBzP	cg02145310	chr10:80844809	*ZMIZ1*	OpenSea	-0.024	0.004	1.95E-07	1.38E-01
BBzP	cg00875096	chr2:196522949	*SLC39A10*	Island	-0.002	0.000	4.65E-07	1.64E-01
MCPP	cg00015121	chr14:24024633	*THTPA*	N_Shore	-0.018	0.003	8.64E-08	6.10E-02
Sex-Interaction^a^								
ΣDINCH	cg07847809	chr10:92197387	*LOC101926942*	OpenSea	0.027	0.004	1.91E-10	1.35E-04
ΣDINCH	cg23603891	chr1:6479628	*HES2*	Island	-0.015	0.002	5.52E-09	1.95E-03
	cg26097711	chr15:64749577		N_Shelf	0.089	0.014	1.13E-08	2.67E-03

a Sex-interaction models were used for ΣDEHTP and ΣDINCH only due to the limited sample size for these phthalates.

**FIGURE 2:**
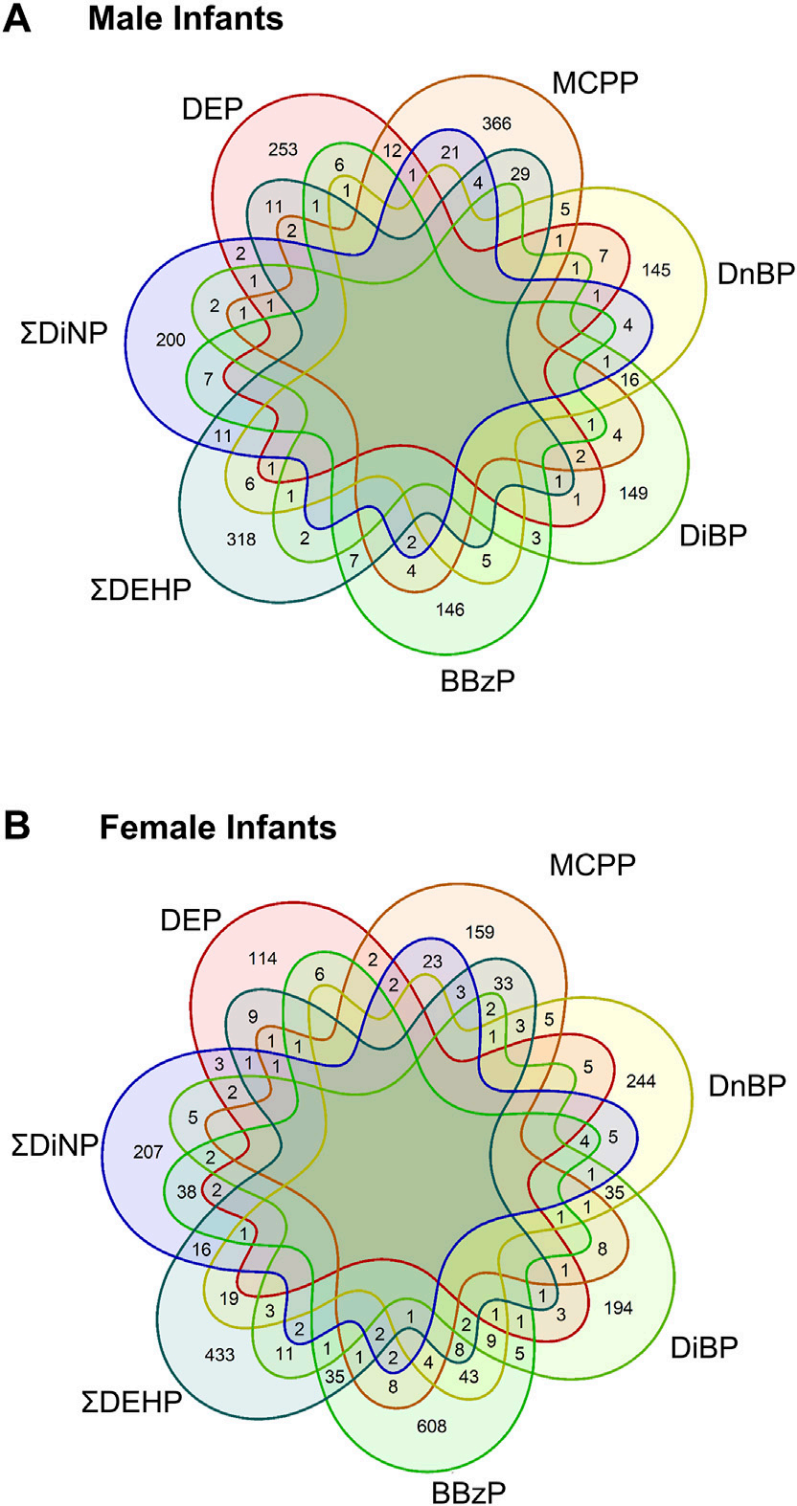
Shared genes in each main model phthalate exposure by infant sex. Venn diagrams of the shared genes in the lists of all genes with a *p*-value of less than 0.001. Top panel **(A)** shows shared genes in males, bottom panel **(B)** showed shared genes in females.

To compare across individual exposures, CpG sites associated with any exposure at a raw *p*-value of <0.001 were extracted from the models. Annotated gene lists from among these CpG sites were compared between main-model exposures, showing that there were no genes shared across all exposures in either sex, and most genes were unique to the individual exposures (Figure 2, see also Supplementary Tables S4, S5). In males, protein tyrosine phosphatase receptor type N2 (*PTPRN2*), a gene involved in secretory processes, was the most commonly shared gene at this cutoff, shared between DEP, ΣDiNP, DiBP, and MCPP. In females, the top-most common genes were also shared in only four phthalates. These included sidekick-1 (*SDK1*), a cell adhesion gene, shared among ΣDEHP, ΣDiNP, DnBP, and BBzP; FAM174A and an olfactory receptor gene (OR10V1) shared among ΣDEHP, ΣDiNP, DiBP, and DnBP; tenascin XB (*TNXB*) and a ribosomal kinase gene (*RPS6KA2*) shared among ΣDEHP, ΣDiNP, BBzP, and MCPP; a brain-specific angiogenesis inhibitor gene (*BAIAP2*) shared among ΣDEHP, DiBP, DnBP, and MCPP; TGFB-induced factor homeobox 1 (*TGIF1*), a transcriptional regulator shared among DiBP, DnBP, BBzP, and MCPP; palladin (*PALLD*), a cytoskeleton organizational gene, shared among ΣDEHP, DEP, ΣDiNP, and MCPP; and PTPRN2 shared among ΣDEHP, DEP, BBzP, and MCPP.

When comparing our results to other epigenome-wide studies of prenatal phthalate exposure (Solomon et al., 2017; Chen et al., 2018; Miura et al., 2021), previously published coefficients for the association between phthalate and DNA methylation at significant CpG sites were not correlated with any phthalates in the present analysis (*p* > 0.05). Because previously reported studies focused on metabolites of only DEHP, we also compared coefficients for specific CpG sites in our ΣDEHP models. Of 263 CpG sites previously reported to be differentially methylated in association with maternal exposure to metabolites of DEHP at birth, ΣDEHP coefficients from the current study were directionally the same in both males and females across 29% of sites (Supplementary Table S5).

### DNA Methylation and MMIP-Only Exposures: Phthalate Alternative Results

Using a subset of MMIP (*n* = 79), regression models were used to assess the relationship between maternal, first-trimester ΣDINCH and ΣDEHTP exposure and DNA methylation in cord blood, including cell type and surrogate variables. Sex-interaction models were initially examined, and regression model lambdas were sufficiently minimized (Supplementary Table S3). In the sex interaction model, one CpG site was hypomethylated and 2 were hypermethylated with ΣDINCH concentrations, whereas no CpG sites was identified as differentially methylated in ΣDEHTP models (*q* < 0.2, Table 3). These included a down-methylated CpG in the *HES2* gene (*β* = -0.015, *q* = 0.002), an up-methylated CpG in a long noncoding RNA gene (*LOC101926942*; *β* = 0.027, *q* = 0.00014), and an up-methylated CpG in an unidentified gene on chromosome 15 (*β* = 0.089, *q* = 0.0027). All of these findings also met a strict cutoff of *p* < 9E-8.

### Ingenuity Pathway Analysis: All Phthalate and Phthalate Alternative Results

Annotated genes from the CpG sites associated with all sex-stratified exposures at a raw *p*-value < 0.001 were imported into IPA (Supplementary Tables S4, S5), revealing several enriched canonical pathways that were differentially regulated in male and female models (*p* < 0.05, *z*-score>|2|), but the total percentage of the genes within the pathway that was enriched was <10% for every pathway (Table 4). Inactivated pathways (related to increased methylation) in males included those of integrin signaling related to ΣDiNP exposure, and cardiac hypertrophy signaling, and MAPK signaling related to MCPP exposure. In females, inhibited canonical pathways included kinetochore metaphase signaling and NAD signaling in association with ΣDEHP exposure and neuregulin signaling associated with ΣDINCH exposure. Fewer pathways were activated (related to decreased methylation) in association to exposure. These included androgen and neutrin signaling (ΣDEHP, females), 14-3-3-mediated signaling (BBzP, females), and senescence and an epithelial growth pathway (DiBP, females).

**TABLE 4:** Significant Canonical Pathways Enriched among CpG Sites Associated with Phthalates at *p* < 0.001.

Exposure	Canonical Pathways	-log_10_ (*p*-value)	*z*-score	% of Genes Enriched in Pathway	Exposure-Associated Genes From the Pathway
Male Infants					
ΣDiNP	Integrin Signaling	2.07	2.0	2.4	*BCAR3, CTTN, HRAS, ITGA7, PPP1CB*
MCPP	Cardiac Hypertrophy Signaling (Enhanced)	1.83	2.3	2.5	*GDPD1, GNA13, HDAC4, IL6R, ITGA6, MAP2K1, MAP3K5, MKNK2, NFATC2, OSM, PDE6B, PRKAR1B, TGFB3*
MCPP	Role of MAPK Signaling in Inhibiting the Pathogenesis of Influenza	1.87	2.0	5.4	*BRAF, MAP2K1, MAP3K5, MYD88*
Female Infants					
ΣDEHP	Kinetochore metaphase Signaling Pathway	1.49	2.0	3.9	*CDC16, KNL1, MAD1L1, MIS12*
ΣDEHP	NAD Signaling Pathway	1.56	2.0	3.5	*PARP15, PIK3CG, POLR2G, PRDM13, SLC29A1*
ΣDEHP	Androgen Signaling	1.85	-2.0	3.6	*CACNA1C, CACNB4, CALM1* (includes others), *GNB5, POLR2G, PRKAR2B*
ΣDEHP	Netrin Signaling	2.00	-2.0	5.6	*ABLIM1, CACNA1C, CACNB4, PRKAR2B*
BBzP	14-3-3-mediated Signaling	2.06	-2.2	6.4	*AKT3, CDKN1B, PDCD6IP, RALB, RASD2, TP73, TRAF2, TUBA8*
DiBP	Senescence Pathway	1.32	-2.2	1.7	*ACVR1, CDKN1A, IKBKB, MTOR, PPP3CA*
DiBP	Regulation of the Epithelial Mesenchymal Transition by Growth Factors Pathway	2.03	-2.0	2.6	*IKBKB, mir-192, MTOR, SHC2, SNAI2*
ΣDINCH	Neuregulin Signaling	1.73	2.0	3.5	*ERBB4, PDPK1, PRKCG, STAT5B*

Significant canonical pathways generated using Tox Analysis from Ingenuity Pathways Analysis (IPA), with annotated genes from the CpG sites, *p* < 0.0001. Above, *p*-values, z-scores, and the percent of the pathway enriched are all values derived from IPA, which uses a Fisher’s exact test to assess relationships of the data in comparison to known pathways. Because these are input with methylation data, a negative z-score would suggest an activated pathway, whereas a positive z-score would suggest an inhibited pathway.

**FIGURE 3:**
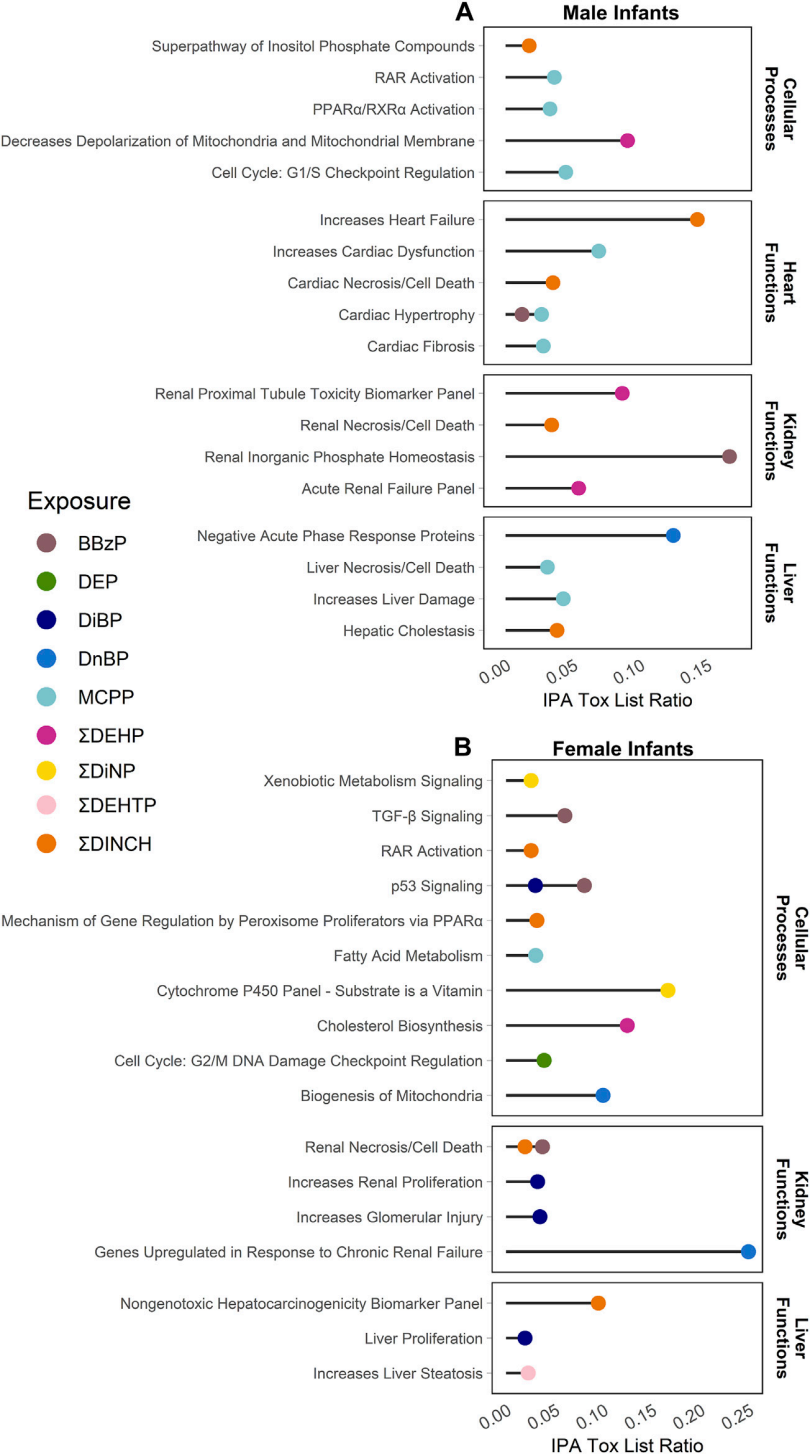
Significantly enriched pathways from the IPA Tox Lists by infant sex. Top panel **(A)** shows results from males, bottom panel **(B)** shows results from females. Using CpGs with a raw *p*-value < 0.001, the % of genes enriched in each pathway are reported by individual phthalate or phthalate alternative. Only human pathways with a *p* < 0.05 from the pathway analysis are included in the figures. Full gene lists can be found in Supplement 6.

**FIGURE 4:**
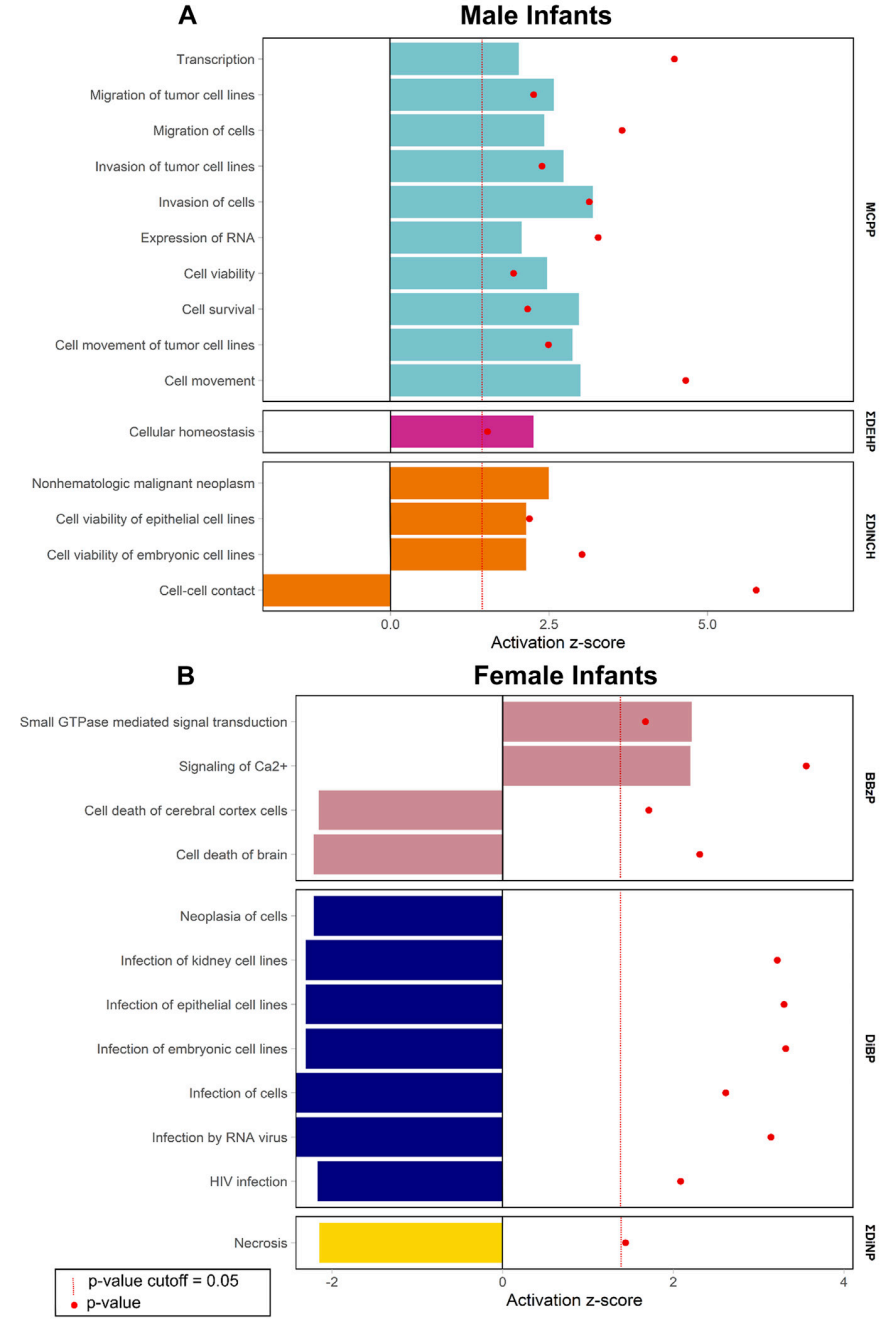
Significantly enriched pathways from the IPA Functions by infant sex. Top panel **(A)** shows results from males, bottom panel **(B)** shows results from females. Using CpG loci with a raw *p*-value < 0.001, the % of genes enriched in each pathway are reported by individual phthalate or phthalate alternative. Only human pathways with a *p* < 0.05 and a *z*-score>|2| from the pathway analysis are included in the figures. Full gene lists can be found in Supplement 6.

Enrichment of pathways in IPA Tox Lists (Figure 3) and IPA functions (Figure 4), were also examined, as these lists are carefully compiled based on published studies on cellular activities and other biological responses. In results from both males and females, Tox Lists were enriched for processes related to responses in the renal and hepatic systems, as well in those processes of general cellular toxicity (Figure 3, *p* < 0.05). Many of the gene lists were similar between males and females, such as those related to essential signaling in pathways related to peroxisome proliferator activated receptor alpha (PPARα) and retinoic X receptor (RXR), but the individual exposure associated with the pathway varied. Males also had enriched pathways related to cardiac functions, whereas females had more cellular processes enriched related to metabolism. IPA Functions were also distinct between exposures and sexes (Figure 4, Supplementary Table S6; *p* < 0.05 and *z*-score> |2|). Enriched pathways in this category were related to MCPP, ΣDEHP, and ΣDINCH for males and BBzP, DiBP, and ΣDiNP for females. In males, all but one function pathway were related to increases in methylation, but, in females, all but two pathways were related to decreases in methylation. Using a random set of genes and corresponding *β* values from the male BBzP model, a similar number of pathways, Tox Lists and functions were significantly enriched (Supplementary Table S7). While several of the Tox Lists did appear to overlap with some of the specific functions, the canonical pathways and IPA functions represented divergent pathways with varying genes.

## Discussion

Phthalates and their increasingly common alternatives are universally found in our daily environments. Over the last decade, mounting evidence has linked exposure to classically used phthalates with a number of significant human health effects. There is a growing body of evidence suggesting that prenatal phthalate exposure is connected with epigenetic effects in developing infants (LaRocca et al., 2014; Wang IJ. et al., 2015; Goodrich et al., 2016; Huen et al., 2016; Solomon et al., 2017; Chen et al., 2018; Huang et al., 2018; Montrose et al., 2018; Tindula et al., 2018; Miura et al., 2021).

The main analysis in this manuscript combined data from two different newborn sample types (blood spots and cord blood) to examine DNA methylation across the genome in relation to maternal exposure to metabolites of six different commonly used phthalates and one nonspecific metabolite (ΣDEHP, DEP, ΣDiNP, DiBP, DnBP, BBzP, and MCPP). This combined cohort approach demonstrated that there was a sex- and exposure-specific relationship with DNA methylation at specific CpG sites, presenting a persuasive piece of evidence of the relationship between DNA methylation and prenatal phthalate exposure. In both male and female models, there were several loci that were suggestive of an association with an individual exposure (*q* < 0.2). These loci were found in genes related general cellular processes, such as transcriptional regulation, as well as more specific process, such as olfaction. One of these loci (*FOXP4* in males) met a stricter significance cutoff of *p* < 9E-8, suggesting an important target gene to consider in future studies. These observed outcomes are particularly salient when considering human epigenomic studies of environmental exposures, as these require large sample sizes to detect subtle effects that are common (Breton et al., 2017). In these studies, even small magnitudes of effects bear close attention when effects are sex specific. Thus, when combined with other results from the literature, these results add evidence to the relationship between prenatal phthalate exposure and epigenetic impacts.

We similarly observed epigenetic associations in the analysis of two common phthalate alternatives. Methylation analyses with our more modest set of mother-infant pairs using sex-interaction models to account for the smaller sample sizes, found several associations with methylation at CpG sites that were related to increasingly common exposures to ΣDINCH, a complex mixture of 9-carbon molecules, rapidly replacing high-molecular weight phthalates (Kasper-Sonnenberg et al., 2019), but not ΣDEHTP, a terephthalate commonly replacing DEHP (Lessmann et al., 2019). While the sample sizes on this analysis were limited, the results underscore the need to closely examine the effects potential replacements before putting them into everyday use to avoid situations of regrettable substitution. Continued focus on these alternatives should better inform the science and policy behind any future use of these compounds.

Pathway analysis can help demonstrate the strength of epigenome-wide studies by revealing overall patterns of differential methylation, and, here, pathways unscored consistent patterns of effects across the literature. Analyses using IPA suggested that prenatal exposure to MCPP in males and ΣDINCH in females were all linked with enrichment in pathways related to PPARα, mirroring results from other human epigenetic studies using targeted approaches (in association with MCPP, BBzP, DEHP) (Montrose et al., 2018). Other research has proposed that phthalates and their metabolites may act through inflammatory pathways (e.g., NF-κB, TNF-β) (Ferguson et al., 2011; Ferguson et al., 2014; Kelley et al., 2019; Nadeem et al., 2020), hormone disruption (e.g., antiandrogenic processes) (Kay et al., 2013; Kay et al., 2014; Axelsson et al., 2015; Montrose et al., 2018), and altered lipid metabolism (e.g., mitochondrial dysfunction) (LaRocca et al., 2014; Benjamin et al., 2017; Braun, 2017; Montrose et al., 2018). Enriched pathways presently echoed these proposed mechanisms, with altered DNA methylation in pathways related to TNF-β signaling (BBzP in females), androgen signaling (ΣDEHP in females), and mitochondrial functions (ΣDEHP in males; DnBP in females). There were not, however, differences in either the methylation of CpG sites or the regulatory pathways related to any key imprinted genes that have previously been reported to be differentially methylated, including *IGF2, H19,* and *MEG3* (LaRocca et al., 2014; Goodrich et al., 2016; Montrose et al., 2018; Tindula et al., 2018), which may be due to differences in EPIC array CpGs and specific CpGs that have been examined in pyrosequencing assays. Pathways were also quite different between infant sex models, once again highlighting the importance of sex-stratified models when it comes to these chemicals. These results, however, should be interpreted in the broader literature context, as IPA selects a single value for any given gene and does not include a penalization term for several CpG sites that are mapped to a given gene (Maksimovic et al., 2021). Given the consistencies within the literature regarding some pathways (e.g., PPARα), we contend that the results presented may still represent important pathway hypotheses to be confirmed with additional sex-stratified and multi-omic studies in the future.

Three other studies on the epigenome-wide effects of prenatal phthalate exposure have also suggested that these toxicants are related to aberrant DNA methylation (Solomon et al., 2017; Chen et al., 2018; Miura et al., 2021). A total of over 300 CpG sites were differentially methylated in association with phthalate exposure in these studies, yet only one study included metabolites outside of those from the parent compound DEHP (Solomon et al., 2017). Contrary to those results, the combined ARCH-MMIP analysis did not present differential methylation in these same CpG loci or genes related to ΣDEHP exposure. Across all of these epigenomic-wide studies of prenatal phthalate exposure, the present combined cohort study was the only to utilize sex-specific methods. Because phthalates are known for sex-specific effects on various outcomes, these differences in analysis approaches may account for some of the dissimilar results across studies (Kay et al., 2013; Kay et al., 2014).

When directly comparing the coefficients from the present sex-stratified models across all parent phthalates to methylation coefficients of previously reported significant CpG sites, methylation coefficients were not correlated. None of the previously reported CpG sites were statistically significant in this present study. When comparing the directional relationship of the male and female DEHP coefficients, over a quarter of the coefficients matched direction in both males and females in the present and previous studies; approximately a quarter matched only in males; another quarter only matched in females; and the last quarter of coefficients from other studies’ CpG sites did not directionally match our coefficients (Supplementary Table S2). Thus, while there were some commonalities in coefficients, coefficient results between studies were somewhat distinct in individual site level differences of DNA methylation. This cross-study comparison highlights the importance of using sex-stratified analyses in phthalate research to understand the subtle effects of these toxicants.

This study was limited from several perspectives, including differences in cohort demographics, sample types, and phthalate assessment methods. In the results, there was minor clustering of the coefficients between cohorts, possibly due to either demographic differences that were unaccounted for in our models or differences in epigenetic signatures in different tissue types. Previous research has shown that the two sample types included here (cord blood and neonatal blood spots) share similar mean methylation at approximately 70% of sites across the epigenome, but correlations across the genome are low (Jiang et al., 2020), suggesting that methylation effects that are unique to certain cell populations may be masked. Results presented in this manuscript attempt to overcome this burden, potentially representing only those genes that are concordantly altered across tissue types.

The scope of this study was limited to only the methylome at birth and did not implicate broader health effects that may be associated with the observed epigenetic associations. To understand the significance of these findings, future studies on developmental epigenetic effects of these compounds should seek to understand the short- and long-term health effects that are linked with both exposure and aberrant DNA methylation (Ponsonby et al., 2016). Additionally, while models included a wide-ranging set of individual parent phthalates, there are differences in biological responses related to both the specific compound of exposure (Meeker et al., 2009) and the timing of exposure during development (Johns et al., 2015; Ferguson et al., 2017). Phthalate exposures vary for any given individual on a day-to-day basis, depending on their behavior, and differential use of individual phthalates in products has strong temporal shifts with consumer preferences and regulation (Zota et al., 2014). Temporality in these exposures may impact the relationship with epigenetic outcomes, while accounting for some of the differences across early-life epigenetic studies discussed above. One study in Mexican American children has even suggested that mid-pregnancy maternal phthalate exposure may be better at predicting methylation differences at birth than exposure data from early pregnancy (Huen et al., 2016). Finally, individual phthalates have differences in mechanisms and potency, and mixture-based assessments may better encompass the compounded effects of phthalates (National Research Council (US) Committee on the Health Risks of Phthalates, 2008).

Despite these limitations, the present research adds to the literature by utilizing two different types of samples to address the sex-specific DNA methylation that is associated with multiple phthalates and phthalate alternative exposures. The comparisons made with other published work and the pathway analyses presented here expand our understanding of this complex relationship by adding additional strength to the evidence of the broader patterns of DNA methylation that are associated with these multiple exposures. Continued examination into how altered DNA methylation in key genes and pathways should be a focus in future research, not only in the case of traditional phthalate exposures, but their widely used alternatives as well.

## Conclusion

Phthalates are an everyday class of chemicals that are challenging to study because of their ubiquitous, but transitory, nature, and the wide-ranging analysis techniques used to assess exposure. This study aimed to overcome some of these barriers by using two neonatal tissue types in two human cohorts to understand how prenatal exposure to seven different parent phthalates is related to epigenome-wide infant DNA methylation at birth. Results demonstrate that there were differences in DNA methylation related to prenatal phthalate exposure, compounding upon mounting evidence that suggests the detrimental effects of these universal compounds. Data presented here also shows concerning evidence that phthalate alternatives may be associated with aberrant epigenetic profiles at birth. As use of these replacement chemicals grow, it is imperative to continue to investigate how prenatal exposure is related to DNA methylation profiles and long-term adverse health outcomes. Scientists and policymakers alike should closely examine the growing of number of studies on phthalates and phthalate alternatives to avoid regrettable substation scenarios in the future.

## Data Availability

The original contributions presented in the study are publicly available. For ARCH, the data can be found on the NCBI GEO database, GSE195595. For MMIP, data are available through the National Institutes of Health Human Health Exposure Analysis Resource (NIH HHEAR) data repository (dois: 10.36043/2273_357, 10.36043/2273_338, and 10.36043/2273_337).
