# Supplementary material for: Prenatal Exposures to Common Phthalates and Prevalent Phthalate Alternatives and Infant DNA Methylation at Birth
Source: Front Genet. 2022 Mar 31;13:793278. doi: 10.3389/fgene.2022.793278 (PMC9010032; doi:10.3389/fgene.2022.793278)
Supplement: Supplementary file 1 [file DataSheet1.docx]

**SUPPLEMENT INFORMATION**

**Supplement 1: Genomic inflation values (lambdas) of main models by infant sex.**

| **Male Infant Model Lambdas** | **ΣDEHP** | **DEP** | **ΣDiNP** | **DiBP** | **DnBP** | **BBzP** | **MCPP** |
| --- | --- | --- | --- | --- | --- | --- | --- |
| Phthalate Only | 4.43 | 13.35 | 4.50 | 3.26 | 4.07 | 21.65 | 2.34 |
| Phthalate + Cell Type + Infant Sex + Maternal BMI + Maternal Income + Surrogate Variables + Cohort Variable | 0.89 | 0.84 | 1.04 | 0.77 | 0.88 | 1.07 | 1.09 |
| **Female Infant Model Lambdas** |  |  |  |  |  |  |  |
| Phthalate Only | 4.57 | 4.57 | 11.60 | 1.29 | 1.35 | 14.53 | 1.63 |
| Phthalate + Cell Type + Maternal BMI + Maternal Income + Surrogate Variables + Cohort Variable | 1.41 | 0.76 | 0.93 | 0.94 | 1.01 | 1.46 | 0.76 |

In the models, CpG site-specific DNA methylation is regressed on only the parent phthalate measurement (log of the raw concentration) or parent phthalate + cell type proportions (granular cells, CD8+ cells, B cells, and nucleated red blood cells) + maternal BMI + maternal income (above or below $50,000 per year) + surrogate variables (representing technical or batch effects) + cohort (ARCH or MMIP)

**Supplement 2: Comparison of DEHP coefficients across all previously reported significant CpGs differentially methylated at birth.**

| **CpG Illumina ID** | **Gene** | **Male ΣDEHP Coefficient** | **Female ΣDEHP Coefficient** | **Coefficient from Other Study** | **Citation** |
| --- | --- | --- | --- | --- | --- |
| **Directionally the Same for Both Males and Females** | | |  |  |  |
| cg22938275 | FKBP10, SC65 | 1.60E-04 | 2.44E-04 | 1.55E-01 | Solomon et al., 2017 |
| cg23823000 | KIF25 | 1.78E-03 | 3.90E-03 | 1.63E-02 | Miura et al., 2020 |
| cg15937073 | HIVEP3 | 3.99E-03 | 1.98E-03 | 1.63E-02 | Miura et al., 2020 |
| cg21913725 | INHBA-AS1 | 1.09E-03 | 7.85E-03 | 1.36E-02 | Miura et al., 2020 |
| cg09613510 | SPOPL | 5.58E-04 | 5.65E-03 | 1.33E-02 | Miura et al., 2020 |
| cg26684601 | JPH3 | 3.39E-04 | 1.30E-03 | 1.30E-02 | Miura et al., 2020 |
| cg12093060 | RAP1GAP | 3.33E-03 | 1.23E-03 | 1.23E-02 | Miura et al., 2020 |
| cg01894508 | ASPRV1 | 2.23E-03 | 2.67E-03 | 1.19E-02 | Miura et al., 2020 |
| cg01143068 | STARD9 | 1.11E-03 | 6.00E-03 | 1.18E-02 | Miura et al., 2020 |
| cg25514475 | UBTD2 | 2.14E-04 | 1.19E-03 | 1.13E-02 | Miura et al., 2020 |
| cg11952340 | USP3 | 7.47E-04 | 3.73E-03 | 1.07E-02 | Miura et al., 2020 |
| cg27366072 | BCL2L14 | 4.45E-04 | 1.54E-03 | 1.06E-02 | Miura et al., 2020 |
| cg09696044 | RBM38 | 3.57E-03 | 5.83E-03 | 9.89E-03 | Miura et al., 2020 |
| cg06828127 | COG2 | 1.00E-04 | 1.60E-03 | 9.80E-03 | Miura et al., 2020 |
| cg13079429 | METTL15 | 2.53E-03 | 2.49E-03 | 9.64E-03 | Miura et al., 2020 |
| cg22904406 | DAXX | 1.95E-03 | 2.87E-04 | 9.59E-03 | Miura et al., 2020 |
| cg14979593 | PGAP2 | 8.09E-04 | 1.50E-04 | 8.90E-03 | Miura et al., 2020 |
| cg00309133 | MIR548H4 | 9.04E-05 | 2.54E-03 | 8.42E-03 | Miura et al., 2020 |
| cg05162166 | ZC3H7B | 1.63E-03 | 1.50E-03 | 7.83E-03 | Miura et al., 2020 |
| cg12543753 | IFFO1 | 5.15E-03 | 2.66E-03 | 7.75E-03 | Miura et al., 2020 |
| cg23473088 | LOC400043 | 1.72E-04 | 2.22E-03 | 7.66E-03 | Miura et al., 2020 |
| cg01001791 | POLR3H | 2.01E-03 | 4.59E-04 | 7.19E-03 | Miura et al., 2020 |
| cg25772723 | GATA2 | 3.61E-04 | 9.68E-04 | 6.96E-03 | Miura et al., 2020 |
| cg09120725 | SMOC2 | 5.61E-04 | 1.63E-03 | 6.88E-03 | Miura et al., 2020 |
| cg15706807 | RP11-513O17.2 | 1.69E-03 | 2.18E-03 | 6.81E-03 | Miura et al., 2020 |
| cg14660328 | MIR5579 | 5.72E-04 | 9.67E-04 | 6.70E-03 | Miura et al., 2020 |
| cg19982668 | MAP3K3 | 5.94E-04 | 4.10E-03 | 6.64E-03 | Miura et al., 2020 |
| cg18075666 | CALD1 | 1.24E-03 | 1.61E-03 | 6.39E-03 | Miura et al., 2020 |
| cg15380263 | FSTL4 | 1.60E-03 | 9.82E-04 | 5.93E-03 | Miura et al., 2020 |
| cg17062665 | E2F7 | 2.45E-03 | 2.60E-03 | 5.75E-03 | Miura et al., 2020 |
| cg02235049 | ADIPOQ | 1.32E-03 | 1.63E-03 | 5.64E-03 | Miura et al., 2020 |
| cg19188855 | HOTAIR | 2.23E-04 | 1.47E-03 | 5.57E-03 | Miura et al., 2020 |
| cg26585320 | FGF9 | 1.84E-03 | 2.14E-03 | 5.42E-03 | Miura et al., 2020 |
| cg09346503 | MIR4262 | 5.63E-04 | 1.07E-03 | 5.28E-03 | Miura et al., 2020 |
| cg27305255 | RERE | 1.56E-03 | 6.51E-04 | 4.99E-03 | Miura et al., 2020 |
| cg00836944 | DAB2IP | 9.88E-06 | 5.50E-04 | 4.75E-03 | Miura et al., 2020 |
| cg00163217 | TUBGCP3 | 1.07E-03 | 3.00E-04 | 4.64E-03 | Miura et al., 2020 |
| cg05353590 | C14orf28 | 4.05E-04 | 1.43E-03 | 4.63E-03 | Miura et al., 2020 |
| cg11697120 | SPON1 | 2.52E-03 | 4.87E-04 | 4.55E-03 | Miura et al., 2020 |
| cg13446622 | SDR9C7 | 7.04E-04 | 2.55E-04 | 4.31E-03 | Miura et al., 2020 |
| cg06496010 | ARID5A | 4.24E-04 | 8.30E-04 | 4.29E-03 | Miura et al., 2020 |
| cg12651645 | PCSK6 | 4.18E-04 | 3.87E-04 | 4.20E-03 | Miura et al., 2020 |
| cg09250965 | HS3ST4 | 8.44E-04 | 1.35E-03 | 4.11E-03 | Miura et al., 2020 |
| cg04134096 | SUCNR1 | 1.61E-03 | 4.93E-04 | 4.07E-03 | Miura et al., 2020 |
| cg09655952 | PROK2 | 1.93E-04 | 3.97E-04 | 3.99E-03 | Miura et al., 2020 |
| cg25233534 | ZNF114 | 9.49E-05 | 5.22E-04 | 3.84E-03 | Miura et al., 2020 |
| cg04772476 | HLA-C | 1.52E-03 | 1.86E-03 | 3.75E-03 | Miura et al., 2020 |
| cg07201017 | FLJ41350 | 1.17E-03 | 7.23E-04 | 3.71E-03 | Miura et al., 2020 |
| cg12157582 | NBPF22P | 3.89E-05 | 7.91E-04 | 3.56E-03 | Miura et al., 2020 |
| cg06053605 | DPYSL2 | 3.13E-04 | 4.69E-04 | 3.25E-03 | Miura et al., 2020 |
| cg21354621 | SFRS2IP | 5.95E-04 | 1.05E-03 | 3.00E-03 | Miura et al., 2020 |
| cg22942101 | ISY1 | 1.40E-04 | 2.18E-04 | 2.98E-03 | Miura et al., 2020 |
| cg07080067 | MRPS36 | 2.52E-04 | 6.27E-04 | 2.92E-03 | Miura et al., 2020 |
| cg24534872 | ARMC7 | 6.14E-04 | 2.13E-04 | 2.92E-03 | Miura et al., 2020 |
| cg26409978 | ZC3H10 | 1.27E-04 | 2.64E-05 | 2.90E-03 | Miura et al., 2020 |
| cg00718831 | IQSEC3 | 4.43E-04 | 3.40E-04 | 2.64E-03 | Miura et al., 2020 |
| cg01472176 | LEFTY1 | 4.82E-05 | 5.74E-04 | 2.44E-03 | Miura et al., 2020 |
| cg10129816 | GPR85 | 6.22E-04 | 7.10E-04 | 2.40E-03 | Miura et al., 2020 |
| cg12979793 | AMDHD1 | 1.70E-04 | 4.06E-04 | 2.40E-03 | Miura et al., 2020 |
| cg19360083 | MGEA5 | 7.19E-05 | 5.72E-04 | 2.25E-03 | Miura et al., 2020 |
| cg14653849 | ATP6V1G1 | 1.86E-04 | 7.20E-04 | 2.08E-03 | Miura et al., 2020 |
| cg03019460 | C11orf51 | 3.17E-04 | 3.27E-04 | 2.04E-03 | Miura et al., 2020 |
| cg25149677 | HSPA13 | 4.51E-04 | 2.42E-04 | 1.89E-03 | Miura et al., 2020 |
| cg14368881 | IFNA2 | 4.77E-06 | 2.86E-04 | 1.73E-03 | Miura et al., 2020 |
| cg23280983 | C2orf42 | 3.98E-04 | 9.74E-04 | 1.70E-03 | Miura et al., 2020 |
| cg03619586 | CLIC1 | 6.92E-04 | 4.64E-04 | 1.39E-03 | Miura et al., 2020 |
| cg19759002 | DRG1 | 9.54E-06 | 2.98E-04 | 1.10E-03 | Miura et al., 2020 |
| cg00935782 | PA2G4 | 3.86E-04 | 1.33E-03 | 1.00E-03 | Chen et al., 2018 |
| cg17244340 | FGD2; FGD2 | 8.91E-04 | 6.27E-04 | 5.00E-04 | Chen et al., 2018 |
| cg06736000 | NA | -2.94E-03 | -1.82E-04 | -9.00E-04 | Chen et al., 2018 |
| cg12611195 | PPP1R14C | -1.00E-03 | -6.89E-04 | -1.20E-03 | Chen et al., 2018 |
| cg20828897 | KCNQ1 | -3.87E-04 | -1.13E-03 | -1.50E-03 | Chen et al., 2018 |
| cg17031607 | PPM1G | -2.37E-04 | -4.10E-04 | -2.36E-03 | Miura et al., 2020 |
| cg00755448 | KCNB2 | -6.55E-04 | -4.13E-04 | -3.09E-03 | Miura et al., 2020 |
| cg00100680 | D4S234E | -2.87E-04 | -1.06E-03 | -4.10E-03 | Miura et al., 2020 |
| cg27657131 | YTHDF1 | -5.23E-05 | -3.51E-04 | -6.92E-03 | Miura et al., 2020 |
| **Directionally the Same for Males Only** | |  |  |  |  |
| cg26562772 | DUSP10 | 1.45E-02 | -7.50E-04 | 1.42E-02 | Miura et al., 2020 |
| cg06972019 | ENO1 | 3.28E-03 | -1.68E-03 | 7.59E-03 | Miura et al., 2020 |
| cg10211414 | RERE | 2.71E-03 | -7.34E-03 | 1.94E-02 | Miura et al., 2020 |
| cg09043226 | AGPAT1 | 2.16E-03 | -9.98E-04 | 3.54E-03 | Miura et al., 2020 |
| cg14397231 | LINC01091 | 2.10E-03 | -2.33E-04 | 9.60E-03 | Miura et al., 2020 |
| cg07630564 | RABJA | 1.78E-03 | -4.98E-03 | 2.10E-03 | Chen et al., 2018 |
| cg12156012 | ARRDC3-AS1 | 1.76E-03 | -6.84E-04 | 6.41E-03 | Miura et al., 2020 |
| cg24692310 | VPS37C | 1.68E-03 | -4.94E-04 | 8.77E-03 | Miura et al., 2020 |
| cg19878200 | TH | 1.65E-03 | -3.38E-03 | 9.24E-03 | Miura et al., 2020 |
| cg00059652 | MAP2K6 | 1.48E-03 | -2.58E-03 | 5.37E-03 | Miura et al., 2020 |
| cg13485533 | ARHGAP24 | 1.42E-03 | -3.66E-04 | 4.40E-03 | Miura et al., 2020 |
| cg16747714 | KRT18; KRT18 | 1.40E-03 | -1.60E-03 | 9.00E-04 | Chen et al., 2018 |
| cg23252587 | FTCDNL1 | 1.37E-03 | -1.60E-04 | 5.06E-03 | Miura et al., 2020 |
| cg15417641 | CACNA1D | 1.34E-03 | -2.49E-03 | 7.82E-03 | Miura et al., 2020 |
| cg11195291 | EDC3; EDC3 | 1.25E-03 | -5.62E-03 | 1.80E-03 | Chen et al., 2018 |
| cg24124069 | CDH23 | 1.09E-03 | -1.22E-03 | 5.46E-03 | Miura et al., 2020 |
| cg12989057 | OLFM4 | 1.09E-03 | -2.71E-03 | 4.21E-03 | Miura et al., 2020 |
| cg16706240 | NCOR2 | 1.08E-03 | -2.01E-03 | 8.34E-03 | Miura et al., 2020 |
| cg09151187 | IPO8 | 9.83E-04 | -1.09E-03 | 5.81E-03 | Miura et al., 2020 |
| cg24329076 | MIR1914 | 9.57E-04 | -1.16E-03 | 7.09E-03 | Miura et al., 2020 |
| cg04421915 | SRGAP3 | 8.42E-04 | -9.68E-04 | 6.12E-03 | Miura et al., 2020 |
| cg24612772 | EXT2 | 7.73E-04 | -1.07E-03 | 2.72E-03 | Miura et al., 2020 |
| cg25328184 | TSLP | 7.47E-04 | -1.41E-04 | 1.69E-03 | Miura et al., 2020 |
| cg22925115 | ACAD9 | 7.36E-04 | -2.69E-03 | 1.79E-03 | Miura et al., 2020 |
| cg08927631 | C5orf33 | 7.33E-04 | -6.68E-04 | 4.46E-03 | Miura et al., 2020 |
| cg25949886 | CD70 | 7.14E-04 | -1.67E-04 | 1.65E-03 | Miura et al., 2020 |
| cg02492207 | MIB2 | 6.99E-04 | -1.76E-03 | 7.56E-03 | Miura et al., 2020 |
| cg19841369 | SYNE2 | 6.91E-04 | -2.88E-03 | 4.32E-03 | Miura et al., 2020 |
| cg15414132 | SLC25A45 | 6.39E-04 | -1.29E-03 | 5.58E-03 | Miura et al., 2020 |
| cg07997434 | UBE2F-SCLY | 5.98E-04 | -1.63E-04 | 4.09E-03 | Miura et al., 2020 |
| cg05149386 | ADM | 5.78E-04 | -3.08E-05 | 3.11E-01 | Solomon et al., 2017 |
| cg24361357 | RREB1 | 5.33E-04 | -2.93E-04 | 1.78E-03 | Miura et al., 2020 |
| cg10929758 | SPTBN1 | 5.00E-04 | -8.66E-04 | 6.31E-03 | Miura et al., 2020 |
| cg13666293 | ZNF721 | 4.71E-04 | -5.04E-04 | 1.49E-03 | Miura et al., 2020 |
| cg08882216 | EPHB6 | 4.47E-04 | -8.69E-04 | 3.34E-03 | Miura et al., 2020 |
| cg14274249 | PLEKHG1 | 4.29E-04 | -1.96E-03 | 5.46E-03 | Miura et al., 2020 |
| cg21786957 | BUD31 | 4.24E-04 | -1.44E-03 | 1.58E-03 | Miura et al., 2020 |
| cg25336892 | CAPN13 | 4.19E-04 | -7.53E-05 | 3.47E-03 | Miura et al., 2020 |
| cg10548708 | ACAA1 | 3.71E-04 | -1.32E-04 | 4.16E-03 | Miura et al., 2020 |
| cg14509895 | TXN | 3.57E-04 | -5.24E-04 | 2.24E-03 | Miura et al., 2020 |
| cg12516504 | CKAP5 | 3.52E-04 | -3.79E-04 | 7.77E-04 | Miura et al., 2020 |
| cg15825692 | WDR63 | 2.98E-04 | -1.28E-03 | 3.95E-03 | Miura et al., 2020 |
| cg21365903 | ADSL | 2.84E-04 | -6.10E-04 | 2.57E-03 | Miura et al., 2020 |
| cg08712866 | GLRB | 2.74E-04 | -1.89E-03 | 1.34E-03 | Miura et al., 2020 |
| cg00647403 | GGA2 | 2.59E-04 | -2.51E-04 | 1.44E-03 | Miura et al., 2020 |
| cg17221864 | WNK2 | 2.22E-04 | -2.96E-04 | 5.62E-03 | Miura et al., 2020 |
| cg27275851 | TBC1D30 | 2.21E-04 | -1.58E-04 | 2.37E-03 | Miura et al., 2020 |
| cg20047489 | MIR124-2 | 2.06E-04 | -4.23E-03 | 1.19E-02 | Miura et al., 2020 |
| cg11212624 | C6orf47 | 1.86E-04 | -5.11E-04 | 2.29E-03 | Miura et al., 2020 |
| cg05932159 | HLA-B | 1.81E-04 | -4.04E-04 | 2.87E-03 | Miura et al., 2020 |
| cg05527267 | SLC43A2 | 1.19E-04 | -4.59E-04 | 6.24E-03 | Miura et al., 2020 |
| cg16829244 | INPP5A | 1.11E-04 | -1.02E-03 | 5.70E-03 | Miura et al., 2020 |
| cg01560642 | TTC34 | 6.41E-05 | -7.66E-04 | 2.90E-03 | Miura et al., 2020 |
| cg08777490 | DUSP4 | 5.81E-05 | -4.63E-04 | 2.13E-03 | Miura et al., 2020 |
| cg10405557 | FCGRT | 3.47E-05 | -6.31E-04 | 2.76E-03 | Miura et al., 2020 |
| cg15606962 | MRPL10 | -8.96E-05 | 6.97E-04 | -2.78E-03 | Miura et al., 2020 |
| cg05921834 | TBCD | -2.26E-04 | 8.18E-04 | -4.34E-03 | Miura et al., 2020 |
| cg12983578 | NA | -4.12E-04 | 2.54E-03 | -1.20E-03 | Chen et al., 2018 |
| cg03046466 | LMF1 | -5.00E-04 | 1.46E-03 | -1.59E-03 | Miura et al., 2020 |
| cg05907939 | PVT1 | -1.42E-03 | 3.34E-03 | -1.10E-03 | Chen et al., 2018 |
| cg16026760 | FEM1A | -1.70E-03 | 1.17E-03 | -2.05E-03 | Miura et al., 2020 |
| cg01880541 | MYOM2 | -2.09E-03 | 4.40E-03 | -1.08E-02 | Miura et al., 2020 |
| **Directionally the Same for Females Only** | | |  |  |  |
| cg04294971 | RFNG | -7.94E-03 | 1.78E-02 | 3.74E-03 | Miura et al., 2020 |
| cg02510924 | RUVBL1 | -4.19E-04 | 6.54E-03 | 5.30E-03 | Miura et al., 2020 |
| cg11191368 | ZNF704 | -7.22E-04 | 5.37E-03 | 5.60E-03 | Miura et al., 2020 |
| cg22524998 | FCHO1 | -1.63E-03 | 4.70E-03 | 8.57E-03 | Miura et al., 2020 |
| cg15232652 | P2RY1 | -7.57E-04 | 4.63E-03 | 8.58E-03 | Miura et al., 2020 |
| cg02735381 | ALPK1 | -1.76E-03 | 4.62E-03 | 4.46E-03 | Miura et al., 2020 |
| cg05947505 | SFXN3 | -2.60E-04 | 4.51E-03 | 9.53E-03 | Miura et al., 2020 |
| cg17684531 | KIAA1688 | -2.19E-03 | 4.27E-03 | 5.17E-03 | Miura et al., 2020 |
| cg20447966 | SFRP1 | -1.56E-03 | 4.11E-03 | 1.27E-02 | Miura et al., 2020 |
| cg00665114 | TNR | -2.68E-03 | 4.02E-03 | 5.90E-03 | Miura et al., 2020 |
| cg17785515 | TOE1 | -9.55E-04 | 3.99E-03 | 4.81E-03 | Miura et al., 2020 |
| cg21186560 | LINC01235 | -6.17E-04 | 3.01E-03 | 1.20E-02 | Miura et al., 2020 |
| cg04314130 | MYT1L | -1.63E-03 | 2.53E-03 | 3.96E-03 | Miura et al., 2020 |
| cg25349939 | GTDC1 | -2.43E-03 | 2.51E-03 | 1.28E-02 | Miura et al., 2020 |
| cg21590729 | JAGN1 | -1.44E-03 | 2.49E-03 | 6.55E-03 | Miura et al., 2020 |
| cg09040174 | IL1F10 | -2.65E-05 | 2.14E-03 | 3.36E-03 | Miura et al., 2020 |
| cg09812623 | HBM | -9.91E-04 | 2.08E-03 | 3.70E-03 | Miura et al., 2020 |
| cg14205001 | NOTCH1 | -8.07E-04 | 1.90E-03 | 8.36E-03 | Miura et al., 2020 |
| cg03665360 | ATP5G2 | -4.88E-04 | 1.83E-03 | 3.57E-03 | Miura et al., 2020 |
| cg10956480 | CACNA1C | -1.00E-03 | 1.71E-03 | 3.80E-03 | Miura et al., 2020 |
| cg16117070 | RHOB | -6.05E-04 | 1.50E-03 | 1.11E-02 | Miura et al., 2020 |
| cg07681561 | CTBP1 | -7.89E-04 | 1.48E-03 | 4.68E-03 | Miura et al., 2020 |
| cg12386808 | ZNF502 | 7.20E-05 | 1.34E-03 | 2.91E-03 | Miura et al., 2020 |
| cg17987649 | UBD | -2.02E-04 | 1.25E-03 | 7.84E-03 | Miura et al., 2020 |
| cg04275566 | ATP11A | -2.59E-04 | 1.06E-03 | 3.09E-03 | Miura et al., 2020 |
| cg22256482 | FBXO32 | -6.47E-04 | 9.92E-04 | 3.45E-03 | Miura et al., 2020 |
| cg13525683 | TIAF1 | -9.16E-04 | 9.43E-04 | 3.96E-03 | Miura et al., 2020 |
| cg12366423 | EGFLAM | -2.06E-03 | 8.09E-04 | 4.96E-03 | Miura et al., 2020 |
| cg12478384 | ADAMTS9 | -3.20E-05 | 8.03E-04 | 1.62E-03 | Miura et al., 2020 |
| cg01330316 | CLIC5 | -1.10E-03 | 7.52E-04 | 4.77E-03 | Miura et al., 2020 |
| cg14298200 | BHLHE22 | -7.28E-04 | 7.35E-04 | 5.44E-03 | Miura et al., 2020 |
| cg22493212 | MIR4277 | -7.03E-04 | 7.00E-04 | 4.31E-03 | Miura et al., 2020 |
| cg09118169 | NUBP2 | -2.84E-05 | 6.93E-04 | 1.32E-03 | Miura et al., 2020 |
| cg07067521 | NUMBL | -1.55E-04 | 6.60E-04 | 2.24E-03 | Miura et al., 2020 |
| cg14848832 | EFNA3 | -3.87E-04 | 6.59E-04 | 3.45E-03 | Miura et al., 2020 |
| cg03994717 | DACT2 | -2.70E-04 | 6.50E-04 | 4.68E-03 | Miura et al., 2020 |
| cg17263823 | TCP10L | -2.25E-04 | 6.26E-04 | 1.52E-03 | Miura et al., 2020 |
| cg08107354 | OLA1 | -5.80E-04 | 5.97E-04 | 9.54E-03 | Miura et al., 2020 |
| cg04465078 | NDE1 | -2.20E-04 | 5.69E-04 | 2.23E-03 | Miura et al., 2020 |
| cg08271804 | ZNF492 | -8.56E-05 | 5.43E-04 | 2.90E-03 | Miura et al., 2020 |
| cg14850660 | HEBP1 | -2.03E-03 | 5.17E-04 | 7.03E-03 | Miura et al., 2020 |
| cg21475281 | C4orf44 | -8.53E-04 | 5.06E-04 | 3.43E-03 | Miura et al., 2020 |
| cg14546394 | MSC | -5.88E-05 | 4.88E-04 | 1.55E-03 | Miura et al., 2020 |
| cg02117713 | CLCN7 | -7.65E-04 | 4.74E-04 | 4.19E-03 | Miura et al., 2020 |
| cg14230647 | PXDN | -1.03E-03 | 4.56E-04 | 7.04E-03 | Miura et al., 2020 |
| cg12437832 | PSMD9 | -2.31E-05 | 3.99E-04 | 2.32E-03 | Miura et al., 2020 |
| cg13680954 | TMEM52 | -1.35E-04 | 3.77E-04 | 2.56E-03 | Miura et al., 2020 |
| cg06685737 | TBX20 | -2.14E-04 | 3.54E-04 | 8.38E-03 | Miura et al., 2020 |
| cg10161039 | IRX3 | -1.52E-04 | 3.23E-04 | 3.25E-03 | Miura et al., 2020 |
| cg19511444 | SOX1 | -1.06E-03 | 2.95E-04 | 2.98E-03 | Miura et al., 2020 |
| cg16701467 | EIF4A1 | -3.82E-04 | 2.84E-04 | 3.11E-03 | Miura et al., 2020 |
| cg02321871 | RCBTB1 | -1.37E-05 | 2.29E-04 | 1.63E-03 | Miura et al., 2020 |
| cg04349178 | PRKCZ | -6.96E-04 | 2.28E-04 | 6.17E-03 | Miura et al., 2020 |
| cg25561140 | MSI1 | -5.06E-05 | 1.75E-04 | 1.66E-03 | Miura et al., 2020 |
| cg06892796 | PTPRN2 | -2.72E-03 | 1.60E-04 | 6.01E-03 | Miura et al., 2020 |
| cg02897989 | PRRX1 | -1.41E-04 | 1.29E-04 | 2.54E-03 | Miura et al., 2020 |
| cg26927516 | LMTK2 | -3.40E-04 | 1.21E-04 | 2.79E-03 | Miura et al., 2020 |
| cg04888491 | TEX29 | -4.50E-04 | 1.09E-04 | 3.03E-03 | Miura et al., 2020 |
| cg05466596 | PACSIN1 | -8.49E-04 | 1.09E-04 | 3.82E-03 | Miura et al., 2020 |
| cg01557215 | DDRGK1 | -1.47E-04 | 7.56E-05 | 2.53E-03 | Miura et al., 2020 |
| cg21491711 | DBN1 | -1.65E-04 | 7.30E-05 | 4.70E-03 | Miura et al., 2020 |
| cg10503037 | EPRS | -9.51E-04 | 5.56E-05 | 4.06E-03 | Miura et al., 2020 |
| cg27366775 | NLRC3 | 2.29E-04 | -3.16E-04 | -1.70E-03 | Chen et al., 2018 |
| cg09973986 | AGAP2 | 1.11E-04 | -4.31E-04 | -1.97E-03 | Miura et al., 2020 |
| cg05265071 | RBPMS2 | 2.25E-04 | -4.65E-04 | -1.30E-03 | Chen et al., 2018 |
| cg07536452 | VAMP2 | 6.63E-04 | -7.75E-04 | -4.32E-03 | Miura et al., 2020 |
| cg14263561 | ZNF618 | 2.55E-03 | -1.78E-03 | -2.01E-02 | Miura et al., 2020 |
| cg19402853 | MFSD2B | 2.92E-03 | -2.18E-03 | -8.00E-04 | Chen et al., 2018 |
| cg21895182 | PPM1L | 7.42E-04 | -2.94E-03 | -8.00E-04 | Chen et al., 2018 |
| cg14961117 | COL13A1 | 1.85E-03 | -5.49E-03 | -9.00E-04 | Chen et al., 2018 |
| **Directionally Opposite for Both Males and Females** | | |  |  |  |
| cg03891644 | LPHN2 | -2.06E-04 | -3.25E-04 | 1.22E-02 | Miura et al., 2020 |
| cg11801481 | FOXA1 | -7.29E-04 | -6.32E-04 | 1.19E-02 | Miura et al., 2020 |
| cg05343289 | SELP | -5.98E-04 | -4.88E-03 | 1.03E-02 | Miura et al., 2020 |
| cg00564857 | SDK1 | -2.34E-03 | -1.03E-03 | 9.43E-03 | Miura et al., 2020 |
| cg03290522 | ARNT2 | -2.05E-03 | -6.61E-04 | 9.37E-03 | Miura et al., 2020 |
| cg07359991 | HSD3B7 | -2.38E-03 | -7.75E-04 | 9.12E-03 | Miura et al., 2020 |
| cg04473654 | SCFD2 | -8.78E-04 | -2.31E-03 | 8.03E-03 | Miura et al., 2020 |
| cg09573515 | RP11-140I24.1 | -1.34E-03 | -4.16E-04 | 7.71E-03 | Miura et al., 2020 |
| cg00672093 | YY1P2 | -2.71E-04 | -1.24E-03 | 7.40E-03 | Miura et al., 2020 |
| cg20893542 | LPP | -3.08E-04 | -1.57E-03 | 7.29E-03 | Miura et al., 2020 |
| cg01518790 | SNAP25-AS1 | -1.52E-03 | -5.36E-04 | 7.24E-03 | Miura et al., 2020 |
| cg25989783 | NCALD | -7.82E-04 | -2.46E-03 | 7.17E-03 | Miura et al., 2020 |
| cg16447680 | KIAA0748 | -4.70E-05 | -1.00E-03 | 6.62E-03 | Miura et al., 2020 |
| cg24701097 | TRIM26 | -2.14E-04 | -8.27E-04 | 6.34E-03 | Miura et al., 2020 |
| cg04217496 | TNFSF11 | -9.50E-04 | -4.12E-04 | 6.15E-03 | Miura et al., 2020 |
| cg26137417 | VARS | -4.50E-03 | -1.05E-03 | 6.14E-03 | Miura et al., 2020 |
| cg24554839 | MIR921 | -5.91E-04 | -1.01E-03 | 6.03E-03 | Miura et al., 2020 |
| cg12719622 | TTC40 | -7.64E-04 | -3.97E-04 | 5.77E-03 | Miura et al., 2020 |
| cg11546554 | OTOS | -1.89E-03 | -1.90E-03 | 5.68E-03 | Miura et al., 2020 |
| cg00606902 | LOC100126784 | -9.70E-04 | -1.23E-04 | 5.27E-03 | Miura et al., 2020 |
| cg06288444 | IGSF9B | -2.10E-04 | -5.97E-04 | 4.91E-03 | Miura et al., 2020 |
| cg19008088 | TLX1 | -1.39E-03 | -2.32E-04 | 4.69E-03 | Miura et al., 2020 |
| cg07826642 | SLC29A1 | -2.34E-04 | -1.27E-03 | 4.67E-03 | Miura et al., 2020 |
| cg00799144 | OSBP2 | -1.01E-04 | -2.19E-03 | 4.63E-03 | Miura et al., 2020 |
| cg27433759 | PIK3CG | -2.08E-03 | -7.64E-04 | 4.58E-03 | Miura et al., 2020 |
| cg10714773 | CECR1 | -1.32E-04 | -2.53E-04 | 4.58E-03 | Miura et al., 2020 |
| cg13653462 | TEX36-AS1 | -9.84E-04 | -7.85E-04 | 4.57E-03 | Miura et al., 2020 |
| cg11530659 | HLA-DQB2 | -2.92E-04 | -1.53E-04 | 4.09E-03 | Miura et al., 2020 |
| cg03078593 | ITPR2 | -3.05E-04 | -2.12E-03 | 4.06E-03 | Miura et al., 2020 |
| cg07410217 | TBC1D1 | -1.26E-04 | -3.18E-04 | 4.05E-03 | Miura et al., 2020 |
| cg01493412 | MYT1L | -8.01E-04 | -8.23E-04 | 4.01E-03 | Miura et al., 2020 |
| cg07002201 | FUT9 | -1.41E-03 | -1.82E-04 | 3.72E-03 | Miura et al., 2020 |
| cg09006420 | VPS53 | -3.89E-04 | -3.10E-05 | 3.63E-03 | Miura et al., 2020 |
| cg18683103 | HABP2 | -2.07E-04 | -2.04E-04 | 3.62E-03 | Miura et al., 2020 |
| cg03819243 | PLCD1 | -2.10E-04 | -1.87E-04 | 3.32E-03 | Miura et al., 2020 |
| cg20152126 | DSE | -1.96E-04 | -2.23E-05 | 3.20E-03 | Miura et al., 2020 |
| cg07792822 | KIRREL | -1.97E-03 | -6.89E-04 | 3.09E-03 | Miura et al., 2020 |
| cg06294712 | BHLHE22 | -3.94E-04 | -6.25E-04 | 2.97E-03 | Miura et al., 2020 |
| cg12291038 | SAMD5 | -5.87E-04 | -2.44E-04 | 2.86E-03 | Miura et al., 2020 |
| cg16501436 | PSMB9 | -1.51E-04 | -1.42E-03 | 2.75E-03 | Miura et al., 2020 |
| cg02743713 | NR4A2 | -1.21E-03 | -2.38E-04 | 2.65E-03 | Miura et al., 2020 |
| cg24745495 | EPHX3 | -1.23E-04 | -9.90E-05 | 2.57E-03 | Miura et al., 2020 |
| cg10820988 | HIAT1 | -3.57E-04 | -3.91E-04 | 2.55E-03 | Miura et al., 2020 |
| cg11182358 | RPA1 | -1.16E-03 | -4.22E-04 | 2.39E-03 | Miura et al., 2020 |
| cg00529371 | GAL3ST2 | -1.67E-04 | -7.04E-04 | 2.27E-03 | Miura et al., 2020 |
| cg09257425 | NT5DC2 | -1.92E-04 | -1.03E-03 | 2.04E-03 | Miura et al., 2020 |
| cg24841008 | EWSR1 | -2.49E-04 | -4.29E-04 | 1.81E-03 | Miura et al., 2020 |
| cg12004730 | TMEM93 | -2.16E-04 | -4.59E-04 | 1.78E-03 | Miura et al., 2020 |
| cg07732421 | HMGCR | -4.86E-04 | -1.96E-04 | 1.50E-03 | Chen et al., 2018 |
| cg23358710 | MRC2 | -1.87E-04 | -6.33E-05 | 8.00E-04 | Chen et al., 2018 |
| cg00764560 | XRCC6; | -5.55E-04 | -1.45E-04 | 8.00E-04 | Chen et al., 2018 |
| cg06090362 | TRIM42; | 1.82E-03 | 3.81E-04 | -7.00E-04 | Chen et al., 2018 |
| cg01882871 | NA | 1.02E-03 | 2.17E-03 | -1.10E-03 | Chen et al., 2018 |
| cg11483884 | TENM2 | 5.53E-05 | 2.29E-03 | -1.30E-03 | Chen et al., 2018 |
| cg21613963 | AKAP8 | 6.23E-04 | 9.71E-05 | -7.62E-03 | Miura et al., 2020 |

CpG sites, genes, and other coefficient columns were extracted from the three previously published epigenome-wide studies on phthalates. Male ΣDEHP Coefficient and Female ΣDEHP Coefficient columns are the matching coefficient estimates for that CpG from the present complex models (see Supplement 1).

**Supplement 3: Genomic inflation values (lambdas) of additional alternative models by sex-interaction model or stratified by infant sex.**

| **Sex-Interaction** | **ΣDINCH** | **ΣDEHTP** |
| --- | --- | --- |
| Exposure Only | 0.73 | 1.97 |
| Exposure + Cell Type + Surrogate Variables | 1.69 | 1.20 |
| **Male Infant Model Lambdas** |  |  |
| Exposure Only | 1.64 | 1.59 |
| Exposure + Cell Type + Surrogate Variables | 1.38 | 0.99 |
| **Female Infant Model Lambdas** |  |  |
| Exposure Only | 1.31 | 1.66 |
| Exposure + Cell Type + Surrogate Variables | 0.89 | 0.90 |

In these models, DNA methylation is regressed on only the parent exposure measurement (log of the raw concentration) or parent exposure + cell type proportions (granular cells, CD8+ cells, B cells, and nucleated red blood cells) + surrogate variables (calculated in R).

**Supplement 4:** CpGs used for IPA pathway analysis in male infants (*p*<0.001). CpG Illumina ID describes the CpG name on the EPIC array. Location describes the chromosomal location of the CpG. CpG position described where in the gene the CpG lies. Gene is the annotated gene name (NA=no gene name). Coefficient represents the association between the exposure and DNA methylation from the complex model described in Supplement 1 or 5.

**Supplement 5:** CpGs used for IPA pathway analysis in female infants (*p*<0.001). CpG Illumina ID describes the CpG name on the EPIC array. Location describes the chromosomal location of the CpG. CpG position described where in the gene the CpG lies. Gene is the annotated gene name (NA=no gene name). Coefficient represents the association between the exposure and DNA methylation from the complex model described in Supplement 1 or 5.

**Supplement 6:** Full List of Genes in IPA Functions (from Figure 4). Significantly enriched pathways from the IPA Functions by infant sex. Using CpG loci with a raw *p*-value<0.001, the % of genes enriched in each pathway are reported by individual phthalate or phthalate alternative. Only human pathways with a *p*<0.05 and a *z*-score>|2| from the pathway analysis are included in the figures. p-values, z-scores, and the percent of the pathway enriched are all values derived from IPA, which uses a Fisher’s exact test to assess relationships of the data in comparison to known functions. Because these are input with methylation data, a negative z-score would suggest an activated pathway, whereas a positive z-score would suggest an inhibited pathway.

**Supplement 7:** Significant pathways, functions, and genes generated from randomly selected gene set using the β values from the male BBzP model. Only human pathways with a *p*<0.05 and a *z*-score>|2| from the pathway analysis are included.
